# Energy Deficit and Factors Associated with Energy Balance during a Combat Deployment in U.S. Army Special Operation Forces Soldiers

**DOI:** 10.3390/nu16183072

**Published:** 2024-09-12

**Authors:** Evan G. Tryon, Nicholas D. Barringer, Harris R. Lieberman, William R. Conkright

**Affiliations:** 1 Nutrition Care Division, Madigan Army Medical Center, Joint-Base Lewis McChord, WA 98433, USA; william.r.conkright.mil@army.mil; 2 Military Nutrition Division, U.S. Army Research Institute of Environmental Medicine, Natick, MA 01760, USA; nicholas.d.barringer.mil@army.mil (N.D.B.); harris.r.lieberman.civ@health.mil (H.R.L.); 3 Office of the Surgeon, U.S. Army Special Operations Command, Fort Bragg, NC 28303, USA

**Keywords:** military, energy intake, dietary intake, energy expenditure, mood states

## Abstract

The purpose of this study was to determine the difference between estimated energy expenditure (EE) and self-reported dietary intake (EI), and factors associated with energy balance in deployed U.S. Army Special Operations Forces (SOF) Soldiers. Methods: Forty-six SOF Soldiers (age: 30.1 ± 3.5 yrs, body mass index: 27.7 ± 4.1 kg/m^2^) completed surveys on demographic data, mission activity characteristics, gastrointestinal issues, ration consumption, resilience, mood state, and dietary intake using a 127-question food frequency questionnaire at the end of a six-month deployment. EE was estimated using a SOF-specific prediction equation with a physical activity factor of 2.1. A paired *t*-test compared reported energy intake (EI) with estimated energy expenditure (EE). Pearson correlations identified significant variables associated with energy balance, which were then incorporated into a multiple linear regression model. The regression analysis included Profile of Mood States (POMS) anger and POMS depression as predictor variables to determine their influence on energy balance. Results: Reported mean EI was 2512 ± 1059 kcal·d^−1^, while estimated mean EE was 5272 ± 525 kcal·d^−1^. The mean energy imbalance was −2854 kcal/d (95% CI: −2655 to −3055, *p* < 0.001), with all participants in negative energy balance (range: −492 to −3813 kcal/d). POMS depression (r = 0.517, *p* < 0.01) and POMS anger (r = 0.363, *p* = 0.020) were associated with energy balance. The regression model was significant (R^2^ = 0.23, F (2, 38) = 7.02, *p* < 0.01), with POMS depression significantly predicting energy balance (β = 50.76, *p* = 0.011). Conclusions: Deployed SOF Soldiers reported high EE and limited EI, which may negatively impact performance. Higher POMS depression scores were associated with lower energy deficits. Future studies should investigate the relationship between mood and energy balance, using direct measures of EI and EE.

## 1. Introduction

United States Army Special Operations Forces Soldiers must complete physically demanding operations in extreme environments, resulting in high energy expenditure [1]. Previous studies using doubly labeled water have reported energy expenditure ranging from 3700–6300 kcal·d^−1^ during training and occupational tasks similar to those performed during deployment [2]. Military studies indicate that service members under-consume energy during field training and deployment, particularly when subsisting on rations for extended periods [3,4]. A study during an 8-day U.S. Marine Corps infantry training reported a deficit of approximately 2400 kcal·d^−1^ and body mass losses of about 4% when reliant on operational rations [5]. Finnish Soldiers lost about 2.5% of body mass during an 8-day infantry training while consuming military rations or rations plus energy-rich protein bars, resulting in body mass loss attributed to high energy expenditure (4800–5600 kcal·d^−1^) and consuming about 58% of the energy provided [6]. Conversely, trainees in the 64-day Special Forces Qualification course increased their energy intake by roughly 2000 kcal·d^−1^ when consuming hot food in the dining facility versus operational rations [7].

Energy balance, or the difference between energy intake and energy expenditure, varies during deployment depending on factors such as assigned duties, length of deployment or task, environment, access to food, and the acceptability and variety of available food [8]. Energy intake is crucial for maintaining energy balance and operational performance. Soldiers often rely on operational rations during deployment, especially in austere locations. Although operational rations, such as the Meal Ready to Eat, are designed to provide adequate nutrition for up to 30 days, studies have shown that relying solely on them for more than ten days can lead to a negative energy balance, where energy expenditure exceeds energy intake [3]. Negative energy balance can lead to detrimental effects on bone mass, immune and endocrine function, lower body strength and power, and body composition, possibly decreasing operational performance [9]. Moreover, negative energy balance and military training have been associated with increased mood disturbances, self-reported depression, and gastrointestinal symptoms [10,11]. Intense physical activity, insufficient caloric intake, inadequate sleep, dehydration, and psychological stress—all common experiences during rigorous military training—are proposed to activate the hypothalamic–pituitary–adrenal axis. The hypothalamic–pituitary–adrenal axis plays a critical role in regulating stress perception, metabolism, and the maintenance of muscle mass [12]. Dysregulation of hypothalamic–pituitary–adrenal axis markers in Survival, Evasion, Resistance, and Escape trainees has been linked to reductions in fat-free mass, cognitive performance, and mood state, which may, in turn, influence energy balance and intake [10]. Thus, assessing energy intake, energy expenditure, and factors associated with energy balance is essential to determine feeding requirements and operational factors affecting energy balance.

Energy expenditure can be estimated with relative accuracy in military personnel using validated procedures. Prediction equations using body mass, body composition, and types and duration of physical activity based on operational demands are highly correlated (r = 0.74–0.76) with energy expenditure measured using the gold standard doubly labeled water technique [2]. While predicting energy expenditure is relatively straightforward, estimating energy intake presents more challenges, especially in military settings. Validated questionnaires, such as the 127-question Block Food Frequency Questionnaire, can assess energy intake and are comparable to weighed food records [13]. Block Food Frequency Questionnaires are frequently used in the military to estimate dietary intake over defined periods and can be used for groups with similar dietary intakes and limited food variety [14,15]. Despite the utility of these tools, there is a lack of published data assessing energy intake and expenditure in deployed Special Operations Forces personnel and predictors of energy balance in Special Operations Forces personnel during real-world deployments.

Given that Special Operations Forces personnel operate in austere environments and may rely on operational rations for extended periods, this is an important area of exploration with potential impacts on operational performance, readiness, susceptibility to illness, and overall health. This study examined the difference between self-reported energy intake and estimated energy expenditure and determined the relationship between Soldier characteristics and energy balance status during Special Operations Forces deployment. Specifically, while previous studies have explored energy balance in military populations, this study’s novelty lies in the fact that data were collected in real-time during actual deployments. This provides a more accurate and ecologically valid representation of the challenges faced by Special Operations personnel, enhancing the relevance of the findings for military operations. To the best of our knowledge, there has been one study that examined physiological markers in Special Forces Operation Soldiers during a deployment [1]. We hypothesized participants would be in an energy deficit and that gastrointestinal distress and negative mood states would be associated with energy balance.

## 2. Methods

### 2.1. Study Design

**Figure 1:**
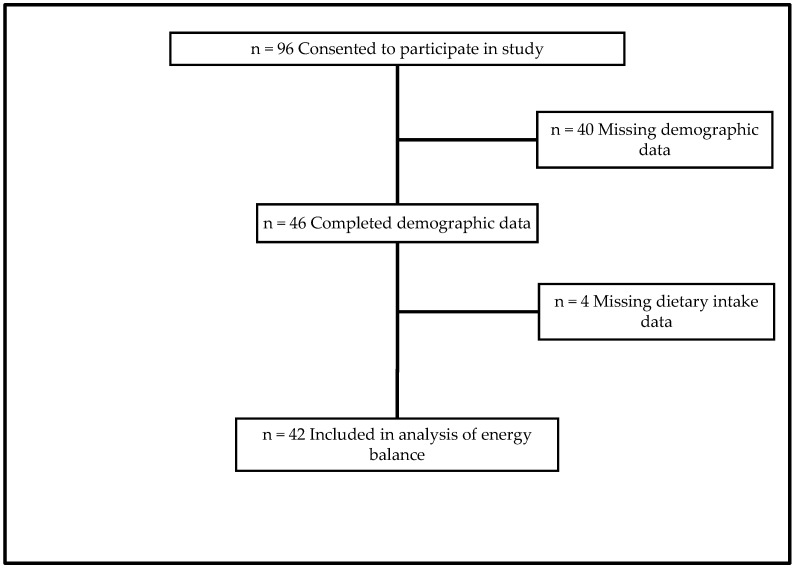
Participant flow and sample size.

The cross-sectional observational study was conducted in theater at the end of a six-month deployment cycle. U.S. Army Special Operations unit Soldiers were involved in this study (male, *n* = 96). We excluded 40 participants for missing key demographic data and four participants for missing dietary intake data. The remaining participants’ (*n* = 46) anthropometrics (body height and body mass), dietary intake (*n* = 42), psychological status, gastrointestinal status, and demographic data were assessed using questionnaires (Figure 1). Questionnaires were administered at the end of the deployment within two weeks of redeployment (i.e., returning to the United States). Surveys were administered in a group setting with no opportunity for discussion. This period was based on the highest anticipated participant availability and lowest mission burden.

### 2.2. Participants

Participants were U.S. Army Special Operations Forces Soldiers who had full medical clearance and were assigned to a U.S. Army Special Operations unit. Soldiers from multiple combat outposts and forward operating bases in the Central Command theater were provided a recruitment brief, and those who provided consent were assigned a participant number to maintain confidentiality. Participation was voluntary and informed consent was obtained from each Soldier before the initiation of data collection. This research was conducted under a Memorandum of Agreement between the U.S. Army Special Operations Command and the U.S. Army Research Institute of Environmental Medicine. The USARIEM Institutional Review Board approved this study.

### 2.3. Procedures

#### 2.3.1. Demographic Data

Demographic information was obtained using a self-reported questionnaire previously used by Special Forces Assessment and Selection trainees and included age, sex, ethnicity, education level, height, weight, race, marital status, sleep duration, supplement use, enlistment status, number of combat deployments, and active-duty time [16]. The demographic questions posed to participants included their sex. While US Army Special Operations units are not exclusively male by policy, this study’s cohort happened to consist entirely of male participants. Combat deployment experience and active-duty time were converted to total months from years and months.

#### 2.3.2. Energy Expenditure

Estimated energy expenditure was calculated using the prediction equation developed by Barringer et al. [2]. This SOF-specific equation is highly correlated (r = 0.74, r^2^ = 0.55) with doubly labeled water assessment of energy expenditure. A physical activity factor (PAF) of 2.1 was chosen based on the intensity and duration of physical activities performed by participants during deployment such as patrols, load carriage, and other mission-specific duties, similar to the demands of Squad Raids and Pre-Mission Training specified by Barringer et al. [2]. Additionally, the PAF aligns with the World Health Organization’s (WHO) recommendations for ‘very active’ individuals and is supported by previous military studies that have employed similar factors to estimate energy expenditure under operational conditions [17].

(Kcal · d − 1) = [47.97 × BM (kg)] + [706.33 × PAF] − 467.22.

#### 2.3.3. Dietary Information

Dietary intake was assessed with a self-administered paper and pencil 127-question Block Food Frequency Questionnaire from NutritionQuest (Berkeley, CA, USA) [18]. The Block Food Frequency Questionnaire required approximately 30 min to complete and estimated the usual intake of food groups and nutrients consumed over the previous six months. Assessment of dietary intake was primarily reflective of the food consumed during deployment. The Block Food Frequency Questionnaire was used to assess how often (never, a few times per six months, once per month, 2–3 times per month, once per week, twice per week, 3–4 times per week, 5–6 times per week, and every day), how much (how much on those days), and what type (e.g., low-fat, low sugar, non-alcoholic, fortified, low carb, whole wheat, etc.) of food was consumed. Macronutrients (total grams of carbohydrate, protein, or fat) and total energy intake (kilocalories) are derived from the frequency and quantity of food items reported on the Block Food Frequency Questionnaire. Each volunteer was provided with a series of pictures developed with the Block Food Frequency Questionnaire to aid in determining the portion sizes while completing the questionnaire. The Block Food Frequency Questionnaire has been validated against multiple diet records [18]. The Block Food Frequency Questionnaire has good volunteer acceptability among Special Forces personnel and is the most frequently used energy intake assessment method in military research [14,19].

#### 2.3.4. Combat Feeding Ration Questionnaire

The Combat Feeding Ration Questionnaire was developed by the United States Army Research Institute of Environmental Medicine (Natick, MA, USA) to assess the frequency of ration consumption, food consumed in the local economy, eating location, and food preferences during missions. A ration reliance composite score was calculated based on the responses to “Please select how frequently you eat the following items in between missions”. Respondents could select from Meal Ready to Eat, Unitized Group Ration, or Other. Meal Ready to Eat rations are individual rations composed of real food items fortified and packaged to meet military-specific nutritional requirements. Unitized Group Rations are a hybrid meal kit designed to feed a group of 50 people that includes perishable or frozen entrees with commercial snack components. Other rations consisted of the Long-Range Patrol Ration, the First Strike Ration, or the Modular Operational Ration Enhancement which are nutritionally incomplete rations specifically designed for high-intensity combat operations and austere environments. Responses were scored on a Likert scale of 0–4 with each selection rated as 1 point (Never = 0, Rarely = 1, Sometimes = 2, Often = 3, Always = 4). The ration composite score could range from 0 to 12, with a higher score indicating higher reliance on rations. Cronbach’s alpha for the internal consistency reliability was determined as acceptable, indicated by α = 0.68 [20].

#### 2.3.5. Gastrointestinal Stress

Gastrointestinal stress was assessed with a self-reported questionnaire that obtained gastrointestinal symptoms, symptom type, and diarrhea episodes to determine the severity of gastrointestinal stress during the deployment. Participants were identified as having experienced gastrointestinal stress by answering yes or no to the following question: “Have you experienced any gastrointestinal symptoms (diarrhea, nausea or vomiting, bloody stool, or abdominal pain) during your current deployment?” A gastrointestinal stress composite score was calculated based on the following question: “What gastrointestinal symptoms have you experienced during your current deployment (Please mark all that apply).” Selections were 1–2 loose or watery stools in 24 h, diarrhea with three or more loose or watery stools in 24 h, blood in stool, vomiting or nausea, moderate to severe abdominal pain unrelated to an injury, and fever. Each symptom was counted as one, and items were summed to derive a score between 0 and 7, with a higher score indicating higher gastrointestinal distress.

#### 2.3.6. Profile of Mood States

Mood states were assessed using the Profile of Mood States, a validated 65-item questionnaire (San Diego, CA, USA) [21]. Participants rate a series of mood-related adjectives on a five-point scale, in response to the question, “How are you feeling right now?” This approach was chosen to capture participants’ immediate emotional states at the final two weeks of deployment, which would likely be influenced by the intense and fluctuating demands of their deployment environment. Understanding these situational factors provides crucial insights into the Soldiers’ psychological resilience and coping mechanisms. The adjectives fall into seven factor-analytically derived mood subscales: tension, depression, anger, vigor, fatigue, confusion, and friendliness. Higher scores for tension, depression, anger, fatigue, and confusion reflect more negative mood states; higher vigor reflects a more positive mood state. Total Mood Disturbance was calculated by summing the totals for the negative subscales and subtracting the total for the positive subscale. A higher Total Mood Disturbance indicates greater mood disturbance. The Profile of Mood States has been used in a wide range of military and civilian populations to assess mood states [10,22,23]. Cronbach’s alpha for the internal consistency reliability was determined as acceptable, indicated by α = 0.65 [20].

#### 2.3.7. Connor–Davidson Resilience Scale

The Connor–Davidson Resilience Scale (Durham, NC, USA) is a validated 25-item self-report questionnaire that assesses responses on a 5-point Likert scale, ranging from 0 (not true at all) to 4 (true nearly all the time) [24]. The scores range from 0 to 100, with a higher score reflecting higher levels of resilience. The questions reflect how the participant felt over the past month.

#### 2.3.8. Statistical Analysis

Statistical Software (SPSS Version 27.0) was used to perform all analyses. Statistical significance for all analyses was set at *p* < 0.05 (two-sided). Descriptive statistics (mean, standard deviation, and percentage) were calculated for all subject characteristics. Shapiro–Wilk, kurtosis or skewness, and residual or regression plots were examined to determine if data were normally distributed. The difference in energy intake and energy expenditure was assessed with a paired *t*-test. A correlation matrix was examined between energy balance and age, weight (kilograms), years of service, number of combat deployments, protein intake (total grams), fiber intake (total grams), carbohydrate intake (total grams), fat intake (total grams), sleep (hours), ration reliance, gastrointestinal composite score, presence of gastrointestinal distress, supplement use, Profile of Mood States subscales and Total Mood Disturbance, and Connor–Davidson Resilience Scale. Variables that were significantly correlated with energy balance (*p* < 0.05), Profile of Mood States anger and Profile of Mood States depression, were entered into a multiple linear regression analysis. Energy balance (intake–expenditure) was the dependent variable, and Profile of Mood States anger and Profile of Mood States depression were the independent variables. Collinearity diagnostics were used to determine multicollinearity with a Variance Inflation Factor of greater than 10 as a cut-off and Pearson’s *r* ≥ 0.8.

## 3. Results

A summary of the physical and anthropometric characteristics of participants is presented in Table 1.

Over one-third of participants reported it was extremely difficult (8.7%) or very difficult (26.1%) to eat during a mission, and 10.9% and 26.1% mostly disagreed and definitely disagreed, respectively, with the statement “I don’t have an appetite when on a mission” (Supplementary Table S1). Additionally, participants mostly agreed (28.3%) and (8.7%) definitely agreed with the statement “I don’t have an appetite when on a mission” with 58.7% of participants agreeing they “didn’t have time to prepare (heat, mix with water) foods when on a mission” and 47.8% of participants agreed that they would “eat more if the rations tasted better,” while only 28.3% agreed they would eat more if they had more food available to them (Supplementary Table S1).

### 3.1. Psychological Variables

The mean (±SD) ration reliance score was 3.1 ± 2.6. The mean Total Mood Disturbance score was 14.3 ± 25.1, and the mean Profile of Mood States subscales were: tension 6.9 ± 5.9; vigor 14.8 ± 6.5; depression 3.7 ± 5.8; fatigue 5.7 ± 5.1; anger 6.8 ± 6.4; confusion 5.9 ± 5.1; and friendliness 12.4 ± 4.8 scores. The mean Connor–Davidson Resilience Scale score was 81.7 ± 11.7. None of the psychological variables demonstrated high collinearity (r < 0.8).

### 3.2. Energy Balance

Reported mean energy intake was 2512 ± 1059 kcal·d^−1^. Estimated mean energy expenditure was 5272 ± 525 kcal·d^−1^. Macronutrient intake distribution was carbohydrate 274.6 g·d^−1^ (~44% of kcal), protein 113.8 g·d^−1^ (1.28 g·kg·d^−1^, ~18% of kcal), and fat 108.0 g·d^−1^ (~38% of kcal). Energy intake and energy expenditure were significantly different, and there was a mean energy imbalance of −2854 kcal·d^−1^ (95% CI: −2655 to −3055, *p* < 0.001). All participants were in negative energy balance (range: −492 to −3813 kcal·d^−1^).

### 3.3. Associations between Demographic, Physiological, and Psychological Characteristics and Energy Balance

Two independent variables were associated with energy balance and included in the regression: Profile of Mood States depression (r = 0.517, *p* < 0.01) and Profile of Mood States anger (r = 0.363, *p* = 0.02; Table 2).

An increase in Profile of Mood States depression increased the likelihood of being in energy balance. Body mass and energy balance experienced multicollinearity (VF > 10); therefore, body mass was not included in the final model. Profile of Mood States depression and Profile of Mood States anger explained 23% of the variance in energy balance (*p* < 0.01; Table 3).

## 4. Discussion

This study determined the estimated energy balance and the factors associated with energy balance in deployed U.S Army Special Operations Forces personnel. Based on self-reported intake and estimated energy expenditure, all U.S. Army Special Operations Forces personnel were in a negative energy balance. Participants with higher Profile of Mood States depression and anger scores had lower energy deficits. Specifically, multiple regression analysis demonstrated a 23% improvement in the prediction utility of energy balance based on mood states (Profile of Mood States depression and anger). While this improvement is statistically significant, its practical significance is also noteworthy. For instance, this improvement could translate to a more accurate identification of soldiers with severe negative mood states, and therefore at risk for significant energy deficits, allowing for earlier intervention and targeted nutritional or psychological support. Integrating psychological assessments into routine health monitoring during deployments may help improve early identification of Soldiers at risk. These findings provide insight into the energy balance status of Special Operations Forces personnel and the potential influence of mood states on energy balance.

Research has previously identified high operational tempo, mission demands, reduced appetite, and inadequate time to eat as contributing factors to negative energy balance [7,8,17]. During deployment, individual variances in energy intake may be greater than individual variance in expenditure. In deployed Royal Marines, within-person variance in energy intake was greater than energy expenditure [8]. Similarly, participants in our study had higher energy intake variance (800–5975 kcal·d^−1^) than energy expenditure (3254–6748 kcal·d^−1^). This suggests that while the demands of the mission (i.e., energy expenditure) remain relatively fixed, increasing energy intake may be a modifiable target to address energy deficits. Consistent with our study (Table 1), energy intakes in SOF personnel during training range from 50 to 80% of expenditure, with time and access to food being the primary causes of reduced intakes [7]. In our study, we were only able to assess energy intake at a single timepoint and it is likely there was substantial variability in energy intake across the deployment period, although our data were consistent with deployment energy intakes taken from multiple timepoints in deployed Royal Marines [8].

Energy deficits in military personnel are attributed to a combination of unwillingness to eat, high energy expenditures, limited food availability, and mission priorities with extensive and demanding training requirements leading to lower energy intakes [2]. It has been proposed that SOF personnel with high energy expenditures (average of ~4567 kcal·d^−1^) can maintain energy balance when there is ample access to hot food [17]. Soldiers during Special Forces Qualification Course decreased their energy deficit by >1600 kcal·d^−1^) when consuming a mix of rations and hot food in the dining facility versus combat rations exclusively [7]. Participants in our study had access to hot, fresh food and had low reliance on rations during deployment (mean score of 3.1 on a scale of 0–12), yet were still in a negative energy balance. This persistent energy deficit highlights another critical issue: insufficient protein intake.

The mean protein intake per kilogram of body weight of our participants is ~1.28 g·kg·d^−1^, which is well below the 1.5–2.0 g·kg·d^−1^ recommended by Pasiakos et al. when military personnel are in an energy deficit [25]. Inadequate protein consumption could exacerbate the loss of lean mass, underscoring the potential need for high-quality protein supplementation, especially when rations are limited [26]. In our study, BMI was utilized as a measure; however, BMI does not differentiate between lean body mass and fat. As a result, while the BMI indicated that participants were ‘overweight,’ this could reflect increased muscle mass rather than excess fat, leading to uncertainty regarding the presumed losses of lean mass and fat mass in this population. In a deployed setting with extensive and critical real-world demands, hot, fresh food alone may not suffice to meet energy and protein needs, necessitating additional nutritional strategies.

In our study, all participants had an energy deficit of >−400 kcal·d^−1^, with some participants having deficits >−2000 kcal·d^−1^. An average daily energy deficit of approximately −100 to −300 kcal·d^−1^ and a corresponding body mass loss of 0.3–3.3% over approximately two months has been associated with minimal physical performance decrements, whereas deficits greater than −928 kcal·d^−1^ for two months were associated with large decrements in lower body performance [27]. In agreement, Royal Marines during an Afghanistan deployment were in a negligible energy deficit (~−143 kcal·d^−1^) and were able to maintain physical performance [8]. The Royal Marines energy intake was assessed via 4- and 7-day food records during the pre-, mid-, and post-deployment period, while energy expenditure was assessed with doubly labeled water [8]. Royal Marines had access to fresh food and field rations, with similar mean energy intakes to our population (~2530 kcal·d^−1^ versus 2512 kcal·d^−1^). Despite similar energy intakes, participants assessed in our study had mean energy expenditures of >5000 kcal·d^−1^ and energy deficit of ~−2500 kcal·d^−1^, similar to studies of energy balance utilizing doubly labeled water in Army Ranger training and the Special Forces Qualification Course [17,28]. This is significantly higher than the Royal Marines sample who had mean energy expenditures of ~3500 kcal·d^−1^. However, participants’ energy expenditure in our study was estimated for the entire deployment and may have been overestimated, leading to exaggerated estimates of energy deficits as participants probably had recovery periods between missions with significantly lower energy deficits and less of an imbalance. Therefore, the contrasts in energy deficits between Soldiers in this study vs. the Royal Marines may reflect the austere environment and physical demands placed on the individual Soldier.

No studies to date have examined the relationship between self-reported mood and energy balance in a deployed military population. In U.S. Marines undergoing Survival, Evasion, Resistance, and Escape training, increases in Total Mood Disturbance and depression scores were associated with decreases in body mass and fat-free mass [10]. Similarly, in a Ranger training exercise designed to simulate combat, degradations in mood including vigor, fatigue, confusion, depression, and tension were associated with significant weight loss of 4.1 ± 0.2 kg (*p* < 0.001) [29]. Notably, both Ranger and Survival, Evasion, Resistance, and Escape trainee energy intake was substantially restricted by course cadre to ~1300 kcal·d^−1^. Combat sport athletes preparing for competition demonstrated that rapid weight loss was associated with greater Total Mood Disturbance and higher anger [30]. Choma et al. hypothesized that mood changes might be a function of decreased body weight, and when there is a return to normal weight, mood stabilizes [31]. It is important to note that POMS subscales, such as depression and anger, are not entirely independent; they often interrelate because mood states can influence each other [21]. For example, heightened anger may correlate with increased depression due to their interconnection, where negative emotions such as anger can contribute to or exacerbate depressive symptoms and vice versa. However, elevated anger scores might reflect specific incidents rather than broader psychological trends. This study assessed this interrelationship using Pearson’s *r* (*r* < 0.8). Future research should use longitudinal mood assessments to better capture sustained mood patterns.

Our study differs from others since higher depression and anger scores were associated with lower energy deficits. Unlike combat athletes undergoing rapid weight loss, energy deficits are not intentional in Soldiers and rather a function of the mission and the environment in which they operate. Smaller energy deficits with higher depression and anger scores may be related to larger energy intakes versus lower expenditures. To test this hypothesis, a post-hoc Pearson correlation and linear regression demonstrated that Profile of Mood States depression was more associated with energy intake (r = 0.474, adjusted r^2^ = 0.205, *p* < 0.01) than energy expenditure (r = 0.404, adjusted r^2^ = 0.144, *p* < 0.01). Therefore, during periods of low control, high stress, and access to hot food, Soldiers may compensate with food as a coping mechanism. During U.S. Navy Sea, Air, and Land Hell Week, energy balance was achieved when trainees had access to four hot meals daily [29]. Sea, Air, and Land trainees have minimal control over their high-stress environment, and despite limited time to eat, they could achieve energy balance. Additionally, Soldiers achieving energy balance while being sleep deprived (4 h per night) during sustained military operations demonstrated decreased self-control and increased risk-taking compared to those in deficits, which was possibly attributed to greater feelings of hunger [32]. Alternatively, in our study, the energy intake and energy expenditure questionnaires were framed to assess the period of deployment, when operations were more frequent, while mood data reflected participants’ current mood (within two weeks of redeployment). At the end of deployment, participants had a lower operational tempo and may have had degraded moods due to not being able to participate in missions. With fewer missions, energy expenditure was likely lower, while access to hot food was greater. Future investigations may need to examine changes in eating behaviors and mood states concerning energy balance to determine if energy balance is a function of access, behavior, or both.

## 5. Limitations

A key strength and limitation of this study is the real-time data collection during actual deployments, which provides a more authentic assessment of the relationship between energy balance, mood disturbances, and gastrointestinal disruptions in the specific conditions of military operations. However, the use of self-reported measures, despite their recognized limitations, was necessitated by the practical constraints of the deployment environment. Data were collected at a single time point (at the end of deployment within two weeks of redeployment), which limits the study’s ability to detect changes over the course of the deployment or establish causality. The reliance on self-reported data through questionnaires introduces potential recall bias. Energy balance was estimated using self-reported height and weight, rather than an objective method that measures fat-free mass. This limitation restricted the precision of participant anthropometrics and the accuracy of the energy balance estimation.

Additionally, mood was assessed using the Profile of Mood States questionnaire, focusing on the ‘right now’ response time frame. These ‘right now’ assessments should be interpreted with caution, as they may reflect transient emotional responses to immediate environmental stimuli rather than underlying mood states. For example, elevated anger scores might be attributed to a specific incident during the mission rather than indicating a broader psychological trend. To account for abrupt changes in mood, the Profile of Mood States questionnaires were administered during a period of very low to no operational requirements, within two weeks of redeployment to the United States. Future studies should consider longitudinal mood assessments to better capture sustained mood patterns.

Combat rations menu items may also be difficult to assess using the Block Food Frequency Questionnaire and could have contributed to underreporting of energy intake. However, reliance on combat rations was low, and units rotated in and out of austere versus more developed locations where they had better access to a broad range of foods. In addition, our reported energy intake aligns with studies in similar settings and with synchronous data collection and measurement tools. Energy expenditure assessment utilized a Special Operations Forces prediction equation and researcher-selected physical activity factor, possibly leading to over or underreporting of expenditure. However, the physical activity factor was based on similar activity demands demonstrated with the prediction model. Regardless of method, estimating energy expenditure is complex, and while doubly labeled water is the gold standard for assessing population risk, it provides mean estimates over 7–14 days and does not account for within-day variability. Our study population consisted of entirely male participants. This limitation should be considered when applying the results to female soldiers in other Special Operations units or in mixed-gender units globally.

## 6. Conclusions

This is the first study to examine the energy balance status of deployed Special Operations Forces Soldiers in a combat zone and the factors associated with energy balance. All Special Operations Forces Soldiers were in a significant energy deficit, which may affect physical and operational performance. Energy balance may be associated with mood states, such as depression and anger. Future longitudinal studies should evaluate the impact of mood states on energy balance and other psychological characteristics to determine the direction and strength of the relationship.

## Figures and Tables

**Table 1:** Summary of the characteristics of participants.

Variable	N	Mean ± SD OR Frequency (%)
Age, years	46	30.3 ± 3.5
Body mass index, kg/m^2^	46	27.8 ± 3.4
Body height, cm	46	179.1 ± 6.6
Combat deployment, # of deployments	46	2.5 ± 1.6
Deployment experience, months	46	20.0 ± 14.0
Service time, years	46	8.9 ± 3.9
Sleep, hours	45	6.8 ± 0.9
Body mass, kg	46	88.8 ± 7.5

kg/m^2^ = kilograms per square meter, cm = centimeters, kg = kilograms, # = number.

**Table 2:** Analysis of the relationship between energy balance and macronutrient intake (grams), ration reliance, presence of gastrointestinal stress, gastrointestinal composite score, combat deployments, total deployment experience, total service experience, age, and sleep (hours).

Variable	Mean (SD)	Correlation Coefficient	*p*-Value
Fat intake (total grams)	107.79 (45.36)	−0.257	0.100
Carbohydrate intake (total grams)	274.57 (130.63)	−0.244	0.119
Protein intake (total grams)	113.80 (47.52)	−0.161	0.308
Fiber intake (total grams)	20.34 (8.57)	−0.220	0.161
Ration reliance	3.13 (2.51)	−0.112	0.481
Presence of gastrointestinal stress (Yes/No)		0.161	0.309
Gastrointestinal composite score	4.15 (3.52)	−0.070	0.660
Combat deployments	2.59 (1.78)	0.168	0.288
Total deployment experience (months)	20.02 (14.05)	0.150	0.342
Total service experience (months)	107.08 (47.08)	0.158	0.318
Age	30.07 (3.50)	0.237	0.131
Sleep (hours)	6.93 (0.94)	0.136	0.398
Profile of Mood States tension	6.87 (5.85)	0.143	0.373
Profile of Mood States depression	3.73 (5.84)	0.517 **	<0.001 **
Profile of Mood States confusion	5.93 (5.08)	0.257	0.105
Profile of Mood States vigor	14.76 (6.48)	−0.002	0.989
Profile of Mood States fatigue	5.73 (5.05)	−0.018	0.912
Profile of Mood States anger	6.82 (6.43)	0.363 *	0.020 *
Profile of Mood States Total Mood Disturbance	14.33 (25.11)	0.299	0.057
Connor Davidson Resilience Scale	82.09 (11.72)	0.084	0.607

* = *p* < 0.05, ** = *p* < 0.01. Significance set at *p* < 0.05.

**Table 3:** Multiple linear regression (enter) of POMS-D and POMS-A on energy balance.

Independent Variable	β	*t*-Value	*p*-Value	Adjusted R Square
Constant	−3099.517	−24.098	<0.001	0.231
Profile of Mood States depression	50.768	2.680	0.011 *	
Profile of Mood States anger	6.61	0.68	0.702	

* = *p* < 0.05. Significance set at *p* < 0.05.

## Data Availability

Data are available per request related to legal issues.

## Supplementary Materials

The following supporting information can be downloaded at: https://www.mdpi.com/article/10.3390/nu16183072/s1, Table S1: Ration Questionnaire Responses.

## Abbreviations

EE	energy expenditure
EI	energy intake
SOF	Special Operations Forces
POMS	Profile of Mood States
