# Supplementary material for: Energy Deficit and Factors Associated with Energy Balance during a Combat Deployment in U.S. Army Special Operation Forces Soldiers
Source: Nutrients. 2024 Sep 12;16(18):3072. doi: 10.3390/nu16183072 (PMC11434678; doi:10.3390/nu16183072)
Supplement: Supplementary file 1 [file nutrients-16-03072-s001.zip › Tryon_SupplementaryTable_S1_Nutrients.pdf]

Supplementary Table S1. Ration Questionnaire Responses

Ration Questionnaire Question	Response (N)	Number of Responses	Percentage (%)
**1. The ration should be easy to eat while on the move *(that is when there is no time to stop to eat).***	Definitely Disagree<br>Mostly Disagree<br>Neither Agree or<br>Disagree<br>Mostly Agree<br>Definitely Agree	1 (2.2)<br>3 (6.5)<br>7 (15.2)<br>17 (37.0)<br>18 (39.1)	
**2. The weight and volume of the ration should be an important factor in its development**	Definitely Disagree<br>Mostly Disagree<br>Neither Agree or<br>Disagree<br>Mostly Agree<br>Definitely Agree	2 4.3<br>8 17.4<br>16 34.8<br>20 43.5<br>46 100.0	
**3. Rations used during training should be the same as used in Wartime.**	Definitely Disagree<br>Mostly Disagree<br>Neither Agree or<br>Disagree<br>Mostly Agree<br>Definitely Agree	3 6.5<br>13 28.3<br>17 37.0<br>7 15.2<br>6 13.0	
**4. Rations taste bad.**	Definitely Disagree<br>Mostly Disagree<br>Neither Agree or<br>Disagree<br>Mostly Agree<br>Definitely Agree	2 4.3<br>7 15.2<br>14 30.4<br>16 34.8<br>7 15.2	
**5. I would eat more on a mission if the rations tasted better.**	Definitely Disagree<br>Mostly Disagree<br>Neither Agree or	3 6.5<br>6 13.0<br>15 32.6	

	Disagree<br>Mostly Agree<br>Definitely Agree	14 30.4<br>8 17.4	
**6. I would eat more on a mission if I had more food available to me.**	Definitely Disagree<br>Mostly Disagree<br>Neither Agree or<br>Disagree<br>Mostly Agree<br>Definitely Agree	3 6.5<br>13 28.3<br>17 37.0<br>7 15.2<br>6 13.0	
**7. I have enough to eat when I am on a mission.**	Definitely Disagree<br>Mostly Disagree<br>Neither Agree or<br>Disagree<br>Mostly Agree<br>Definitely Agree	4 8.7<br>18 39.1<br>21 45.7<br>3 6.5	
**8. I cannot carry all of the food that I need when I go on a mission.**	Definitely Disagree<br>Mostly Disagree<br>Neither Agree or<br>Disagree<br>Mostly Agree<br>Definitely Agree	7 15.2<br>15 32.6<br>13 28.3<br>9 19.6<br>2 4.3	
**9. Foods I like don't taste good when I am on a mission.**	Definitely Disagree<br>Mostly Disagree<br>Neither Agree or<br>Disagree<br>Mostly Agree<br>Definitely Agree	14 30.4<br>14 30.4<br>18 39.1<br>0 0<br>0 0	
**10. I have enough time to eat when on a mission.**	Definitely Disagree<br>Mostly Disagree<br>Neither Agree or<br>Disagree	4 9.1<br>12 27.3<br>15 34.1<br>12 27.3<br>1 2.3	

	Mostly Agree<br>Definitely Agree		
**11. I would eat more on a mission if the rations had more variety.**	Definitely Disagree<br>Mostly Disagree<br>Neither Agree or<br>Disagree<br>Mostly Agree<br>Definitely Agree	2 4.3<br>8 17.4<br>20 43.5<br>12 26.1<br>4 8.7	
**12. I get enough to eat when I am on a mission.**	Definitely Disagree<br>Mostly Disagree<br>Neither Agree or<br>Disagree<br>Mostly Agree<br>Definitely Agree	3 6.5<br>8 17.4<br>15 32.6<br>17 37.0<br>3 6.5	
**13. I consume more caffeine than usual when on a mission.**	Definitely Disagree<br>Mostly Disagree<br>Neither Agree or<br>Disagree<br>Mostly Agree<br>Definitely Agree	3 6.5<br>5 10.9<br>9 19.6<br>12 26.1<br>17 37.0	
**14. I am able to get foods in local communities when on a mission.**	Definitely Disagree<br>Mostly Disagree<br>Neither Agree or<br>Disagree<br>Mostly Agree<br>Definitely Agree	13 28.3<br>12 26.1<br>17 37.0<br>4 8.7	
**15. When on a mission I eat while doing other tasks.**	Definitely Disagree<br>Mostly Disagree<br>Neither Agree or<br>Disagree<br>Mostly Agree<br>Definitely Agree	3 6.5<br>7 15.2<br>6 13.0<br>20 43.5<br>10 21.7	

**16. I barter with ration items when on a mission.**	Definitely Disagree<br>Mostly Disagree<br>Neither Agree or<br>Disagree<br>Mostly Agree<br>Definitely Agree	13 28.9<br>7 15.6<br>13 28.9<br>7 15.6<br>5 11.1	
Please select how frequently you eat the following items *in between* mission?<br>17. MRE	Never<br>Rarely<br>Sometimes<br>Often<br>Always	21 45.7<br>10 21.7<br>8 17.4<br>5 10.9<br>2 4.3	
Please select how frequently you eat the following items in between mission?<br>18.UGR (Unitized Group Ration)	Never<br>Rarely<br>Sometimes<br>Often<br>Always	31 68.9<br>6 13.2<br>1 2.2<br>3 6.7<br>4 8.9	
Please select how frequently you eat the following items *in between* mission?<br>19. Other Rations	Never<br>Rarely<br>Sometimes<br>Often<br>Always	23 51.2<br>2 4.5<br>5 11.4<br>6 13.6<br>8 18.2	
[redacted]			
50. How easy or difficult is it to eat during a mission?	Extremely Difficult<br>Very Difficult<br>Neutral<br>Very Easy<br>Extremely Easy	4 8.7<br>12 26.1<br>26 56.5<br>4 8.7	
53. I am able to eat enough on a mission.	Definitely Disagree<br>Mostly Disagree	5 10.9<br>12 26.1	

	Neither Agree or Disagree<br>Mostly Agree<br>Definitely Agree	14 30.4<br>12 26.1<br>3 6.5	
54. I don't have an appetite when on a mission.	Definitely Disagree<br>Mostly Disagree<br>Neither Agree or Disagree<br>Mostly Agree<br>Definitely Agree	2 4.3<br>15 32.6<br>12 26.1<br>13 28.3<br>4 8.7	
55. Eating is not important when I am on a mission	Definitely Disagree<br>Mostly Disagree<br>Neither Agree or Disagree<br>Mostly Agree<br>Definitely Agree	11 23.9<br>13 28.3<br>10 21.7<br>10 21.7<br>2 4.3	
56. I don't have time to prepare (heat, mix with water) foods when on a mission	Definitely Disagree<br>Mostly Disagree<br>Neither Agree or Disagree<br>Mostly Agree<br>Definitely Agree	0 0<br>7 15.2<br>12 26.1<br>13 28.3<br>14 30.4	
57. I bring additional food items when on a mission	Definitely Disagree<br>Mostly Disagree<br>Neither Agree or Disagree<br>Mostly Agree<br>Definitely Agree	0 0<br>2 4.3<br>4 8.7<br>17 37.0<br>22 47.8	
61. How many times is hunger a distraction from the mission during deployment?	Never/Rarely<br>Once per month<br>2-3 times per month<br>Once per week	25 54.3<br>5 10.9<br>5 10.9<br>1 2.2	

	1-2 times per week<br>3-6 times per week<br>Once per day<br>2 or more times per day	5 10.9<br>1 2.2<br>2 4.3<br>2 4.3	
62. How would you describe your typical hunger level when on a mission?	I am rarely hungry<br>I am hungry some of the time<br>I am hungry most of the time<br>I am hungry all of the time	10 21.7<br>21 45.7<br>13 28.3<br>2 4.3	
63. How often does hunger interfere with your physical or mental performance on a mission?	Never<br>Rarely<br>Sometimes<br>Often<br>Always	12 26.1<br>22 47.8<br>9 19.6<br>3 6.5<br>0 0	
64. I have difficulty concentration on a mission because of hunger	Definitely Disagree<br>Mostly Disagree<br>Neither Agree or Disagree<br>Mostly Agree<br>Definitely Agree	25 54.3<br>12 26.1<br>5 10.9<br>3 6.5<br>0 0	
65. I am hungrier than usual when I return from a mission	Definitely Disagree<br>Mostly Disagree<br>Neither Agree or Disagree<br>Mostly Agree<br>Definitely Agree	1 2.2<br>3 6.5<br>16 34.8<br>6 13.0<br>0 0	
